# Crenarchaeal CdvA Forms Double-Helical Filaments Containing DNA and Interacts with ESCRT-III-Like CdvB

**DOI:** 10.1371/journal.pone.0021921

**Published:** 2011-07-08

**Authors:** Christine Moriscot, Simonetta Gribaldo, Jean-Michel Jault, Mart Krupovic, Julie Arnaud, Marc Jamin, Guy Schoehn, Patrick Forterre, Winfried Weissenhorn, Patricia Renesto

**Affiliations:** 1 CNRS-EMBL-UJF, Unit of Virus Host Cell Interactions (UVHCI)-UMI 3265, Grenoble, France; 2 CEA-CNRS-UJF, Institut de Biologie Structurale Jean-Pierre Ebel, UMR 5075, Grenoble, France; 3 Biologie Moléculaire du gène chez les Extrêmophiles (BMGE), Institut Pasteur, Paris, France; 4 Univ Paris-Sud, Institut de Génétique et Microbiologie, CNRS UMR 8621, Orsay, France; Max-Planck-Institute for Terrestrial Microbiology, Germany

## Abstract

**Background:**

The phylum Crenarchaeota lacks the FtsZ cell division hallmark of bacteria and employs instead Cdv proteins. While CdvB and CdvC are homologues of the eukaryotic ESCRT-III and Vps4 proteins, implicated in membrane fission processes during multivesicular body biogenesis, cytokinesis and budding of some enveloped viruses, little is known about the structure and function of CdvA. Here, we report the biochemical and biophysical characterization of the three Cdv proteins from the hyperthermophilic archaeon *Metallospherae sedula*.

**Methodology/Principal Findings:**

Using sucrose density gradient ultracentrifugation and negative staining electron microscopy, we evidenced for the first time that CdvA forms polymers in association with DNA, similar to known bacterial DNA partitioning proteins. We also observed that, in contrast to full-lengh CdvB that was purified as a monodisperse protein, the C-terminally deleted CdvB construct forms filamentous polymers, a phenomenon previously observed with eukaryotic ESCRT-III proteins. Based on size exclusion chromatography data combined with detection by multi-angle laser light scattering analysis, we demonstrated that CdvC assembles, in a nucleotide-independent way, as homopolymers resembling dodecamers and endowed with ATPase activity *in vitro*. The interactions between these putative cell division partners were further explored. Thus, besides confirming the previous observations that CdvB interacts with both CdvA and CdvC, our data demonstrate that CdvA/CdvB and CdvC/CdvB interactions are not mutually exclusive.

**Conclusions/Significance:**

Our data reinforce the concept that Cdv proteins are closely related to the eukaryotic ESCRT-III counterparts and suggest that the organization of the ESCRT-III machinery at the Crenarchaeal cell division septum is organized by CdvA an ancient cytoskeleton protein that might help to coordinate genome segregation.

## Introduction

The Archaea constitut*e* one of three domains of life, along with Eukarya and Bacteria [1]. Although Archaea resemble Bacteria in terms of size, cell structure and genome organization, they are often much more similar to Eukarya at the molecular level. This is true both for informational mechanisms, such as replication, transcription and translation, as well as for operational mechanisms including ATP synthesis, membrane remodeling systems and production and secretion of membrane proteins [2], [3]. While a specific evolutionary relationship between the Archaea and the Eukarya is generally admitted, its precise nature remains controversial [4]. The Archaea have been historically divided into two major phyla, the Euryarchaeota and the Crenarcheaota. Intriguingly, they exhibit critical differences in some major molecular mechanisms including DNA replication and cell division [5].

Concerning cell division, Euryarchaeota harbour homologues of the bacterial division proteins FtsZ and MinD, whereas these proteins are missing from Crenarchaeota [6], [7], [8]. From genome-wide transcription analysis of synchronized *Sulfolobus acidocaldarius* cultures, a cluster of three genes that are induced at the onset of genome segregation and division were recently identified [9]. These genes encode three proteins (dubbed CdvA, CdvB and CdvC for Cell division), that co-localize at mid-cell during cell division, forming band-like structures between segregating nucleoids [9], [10]. Importantly, CdvB and CdvC are homologous to eukaryotic proteins of the “endosomal sorting complex required for transport (ESCRT)” machinery [11], [12]. In Eukarya, the ESCRT machinery is composed of the complexes ESCRT-0, -I, -II, -III and vacuolar protein sorting (Vps4), that play essential roles in several fundamental cellular pathways including the biogenesis of multivesicular bodies [13], [14], [15], enveloped virus budding [16], [17], [18] and notably cytokinesis [18], [19], [20], [21]. CdvB, encoded by Saci_1373 in *S. acidocaldarius* is an homologue of ESCRT-III, while CdvC (Saci_1372) is homologous to the AAA-type Vps4-like ATPase [9], [10], [11], [12] that is involved in recycling of ESCRT-III [22]. The C-terminus of the archaeal CdvB contains a MIT domain interacting motif (MIM2) that interacts with CdvC [10], similar to the eukaryotic Vps4-MIT-CHMP6 interaction [23]. Consistent with eukaryotic ESCRT function, overexpression of a catalytically inactive CdvC (Vps4) mutant in *Sulfolobus* resulted in the accumulation of enlarged cells, indicative of failed cell division [10]. Together, these results suggest that cell division in *Sulfolobus* relies on an eukaryotic-like ESCRT-III membrane remodeling machinery.

The mechanisms of cell division in this archaeon also exhibit unique features. While CdvB and CdvC resemble the minimal eukaryotic membrane fission machinery [14], [17], the third member of the Cdv cluster, namely CdvA, is only present in Archaea [24]. Potential similarity was reported between CdvA and some components of the eukaryotic cytoskeleton and nuclear envelope like eukaryotic lamins, golgins and cingulin-like proteins [9]. However, these similarities might only reflect the presence of a coiled-coil domain in CdvA. In a recent work, it was shown that the C-terminal domain of CdvA interacts with the C-terminal winged-helix-like (wH) domain of CdvB, a phenomenon that could promote CdvB recruitment to membrane during cell division [25].

Interestingly, a lineage of Crenarchaeota (Thermoproteales) lacks both FtsZ and ESCRT-III homologues and potentially relies on an actin-like based system for cell division [24]. Moreover, Thaumarchaeota, a third major archaeal phylum that has been recently proposed, harbor homologues of both euryarchaeal FtsZ and crenarcheal Cdv systems [5], [9]. These findings raise fascinating questions regarding the evolutionary history of cell division in Archaea. A prerequisite to decipher this complex history is to get a clear picture of all involved molecular machines. However, in the case of the ESCRT-III-based machinery, understanding of the archaeal system is presently hampered by our lack of knowledge on the biochemistry of the Cdv proteins.

Here, we report the biochemical and biophysical characterization of the three Cdv proteins from the hyperthermophilic archaeon *Metallosphaera sedula*, a member of the order Sulfolobales. To explore the putative interactions between the different partners we first established protocols allowing expression and purification of soluble proteins. This led us to show for the first time that CdvA polymerizes into two stranded intertwined filaments containing DNA, which resemble bacterial DNA partitioning proteins. Our data also indirectly provided evidence for a structural similarity between archaeal CdvB and the eukaryotic ESCRT-III proteins and showed that the ATPase CdvC (Vps4) associates into enzymatically active dodecameric-like structures. Finally, the interactions between Cdv proteins were investigated by sucrose gradient centrifugation. These data provide new insights into the functional role of the Cdv proteins during membrane fission in Archaea.

## Results

### Purification of *M. sedula* Cdv proteins

**Figure 1:**
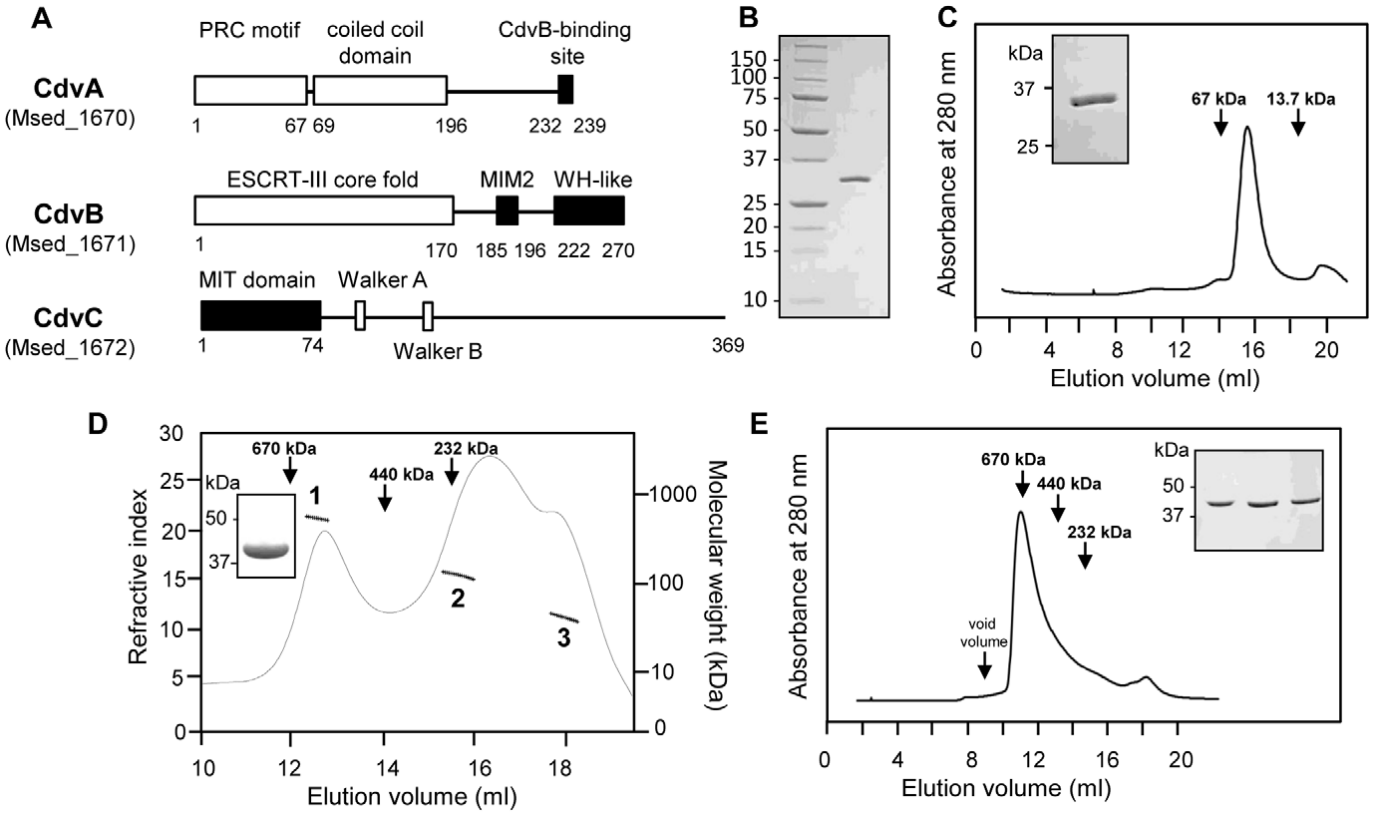
Purification of the Cdv proteins from *M. sedula*. (A) Schematic representation of the proteins encoded by the *cdv* gene cluster from *M. sedula*. The black boxes correspond to protein-protein interaction motifs, i.e the CdvB-binding site of CdvA which interacts with the WH domain of CdvB and the MIM2 of CdvB that binds the MIT domain of CdvC [10], [25]. (B) Recombinant full length CdvA from *M. sedula* was separated by SDS-PAGE (12%) and stained with Coomassie blue. Values on the left indicate molecular weights of the marker proteins. (C) Elution profile of CdvB from a Superdex 200 SEC column. The inset shows the SDS page band of purified CdvB. (D) The SEC profile (Superose 6) of CdvC (50 µM) and MALLS analysis; the inset shows the SDS-page band of CdvC. Protein elution from the column was monitored by refractometry (solid line) and the molar mass was determined by static light scattering (dots). Three different populations were observed. Pic 1: 530±50 kDa; Pic 2: 130±20 kDa; Pic 3: 40±5 kDa. (E) SEC elution profile (Superose 6) of CdvC (180 µM). Inset: Fractions corresponding to the peak separated by SDS-PAGE. The columns were standardized with the following molecular weights markers : blue dextran (2.10^6^ kDa); tyroglobuline (670 kDa); ferritine (440 kDa); catalase (232 kDa); bovine serum albumin (67 kDa) and ribonuclease A (13.7 kDa).

Screening for optimized expression and purification of the three Cdv proteins encoded by the *cdv* gene cluster from *M. sedula* (Figure 1A) revealed that the solubility of the N-terminal His-tagged CdvA could be significantly improved in presence of L-Arginine and L-Glutamine (optimal at 50 mM) in the solubilization buffer, as noted for other proteins [26]. This produced pure CdvA (Figure 1B) which, however, eluted from a Superdex 200 column in the void volume indicating the formation of high molecular weight structures. In contrast, and as judged by size exclusion chromatography (SEC) using low salt buffer conditions (50 mM NaCl), CdvB was purified as a monomeric protein (Figure 1C). The same buffer conditions were applied for CdvC purification. As shown by SEC combined with detection by multi-angle laser light scattering (SEC-MALLS), CdvC elutes in different peaks from the SEC column corresponding to monomers, dimers and probably dodecamers (Figure 1D). The SEC profile also indicated that, at a protein concentration >75 µM, CdvC elutes as a single high molecular weight peak (Figure 1E) corresponding to dodecameric-like homopolymers. The formation of CdvC oligomers occurred in a concentration-dependent manner, which does not depend on the presence of nucleotides. Thus, the oligomerisation was also observed by SEC analysis (Figure S1) when the recombinant protein was treated by ammonium sulfate precipitation in order to remove nucleotides that might have been co-purified.

### CdvA forms double helical filaments stabilized by DNA

**Figure 2:**
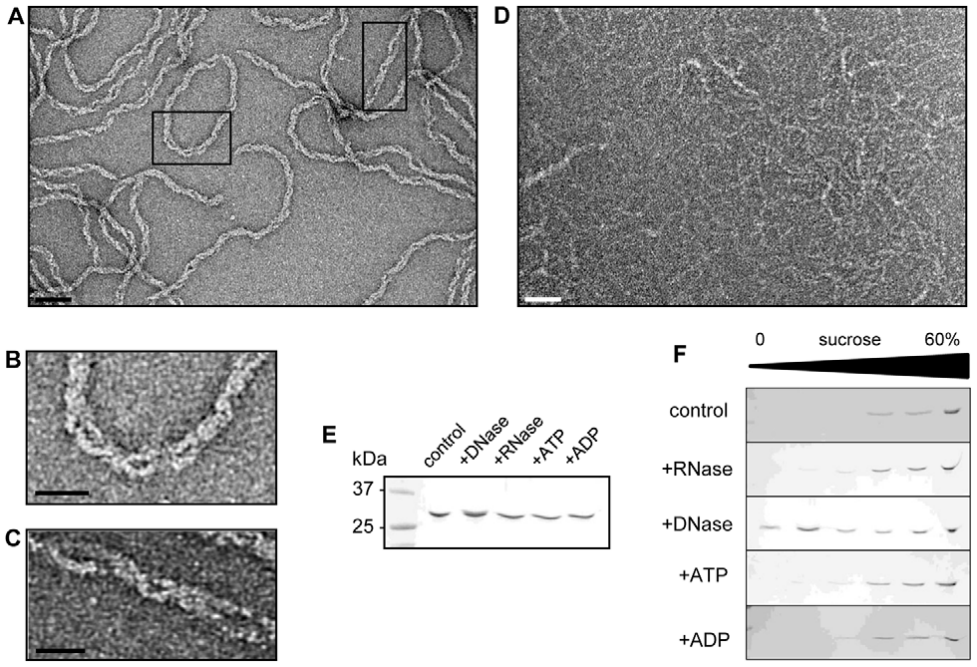
CdvA forms helical filaments stabilized by DNA. (A) Negative staining EM image of CdvA filaments (scale bar, 50 nm). (B, C) Magnifications of the areas marked by black squares in A (scale bars, 20 nm). (D) Negative staining EM micrograph of CdvA pretreated with DNase (scale bar, 50 nm). (E) SDS-PAGE of CdvA incubated for 30 min at 37°C with DNase (20 µg/ml), RNase (20 µg/ml), ATP-MgCl_2_ (1 mM) or ADP-MgCl_2_ (1 mM). (F) Protein distribution of the same samples after discontinuous sucrose density gradient centrifugation.

Negative staining electron microscopy (EM) analyses showed that CdvA present in the SEC void volume peak assembled into two or sometimes three intertwined filaments (Figures 2A, B and C). Their diameter ranges from 80 to 110±3 Å. The OD_260/A280_ ratio (>1) of purified CdvA suggested that polymeric CdvA contains substantial amounts of nucleic acids. In order to determine the nature of nucleic acids associated with CdvA, the polymers were incubated with DNase, RNase, ATP or ADP. As indicated by negative staining EM, DNase affected the polymers because the CdvA filaments changed their appearance following such treatment (Figure 2D) while no change occurred with other compounds (Figure S2). These modifications were not induced by protein degradation, as indicated by SDS-PAGE analysis (Figure 2E). The effect of DNAse on the polymers was further confirmed by sucrose gradient density centrifugation analyses (Figure 2F). CdvA polymers are mainly found in the bottom of the gradient corresponding to their large molecular weight; in contrast, DNAse treatment relocated a substantial amount of CdvA to the upper lower molecular weight protein containing fractions of the gradient, although some large complexes are still observed. Subsequent trials to purify CdvA under denaturing conditions failed to obtain DNA-free CdvA, which would have allowed us to reconstitute CdvA polymers with either single stranded or double stranded DNA. Our data thus indicate that CdvA forms filaments which contain DNA that is derived from the *E. coli* expression host cell.

### CdvA is an ancient protein in Archaea

Homology searches against the nr and environmental databases at the NCBI confirmed that CdvA homologues are present only in Crenarchaeota (except Thermoproteales) and in Thaumarchaeota, and strictly in single copy. All these homologues show a high conservation in sequence and size. Phylogenetic analysis (Figure S3) led to a tree that is remarkably consistent with accepted relationships within Archaea [5], indicating that the distribution of CdvA is not due to recent horizontal gene transfer (HGT). This suggests that CdvA is an ancient archaeal protein that was lost in the Thermoproteales and possibly also in Euryarchaeota.

### CdvA interacts with CdvB but not with CdvC *in vitro*

**Figure 3:**
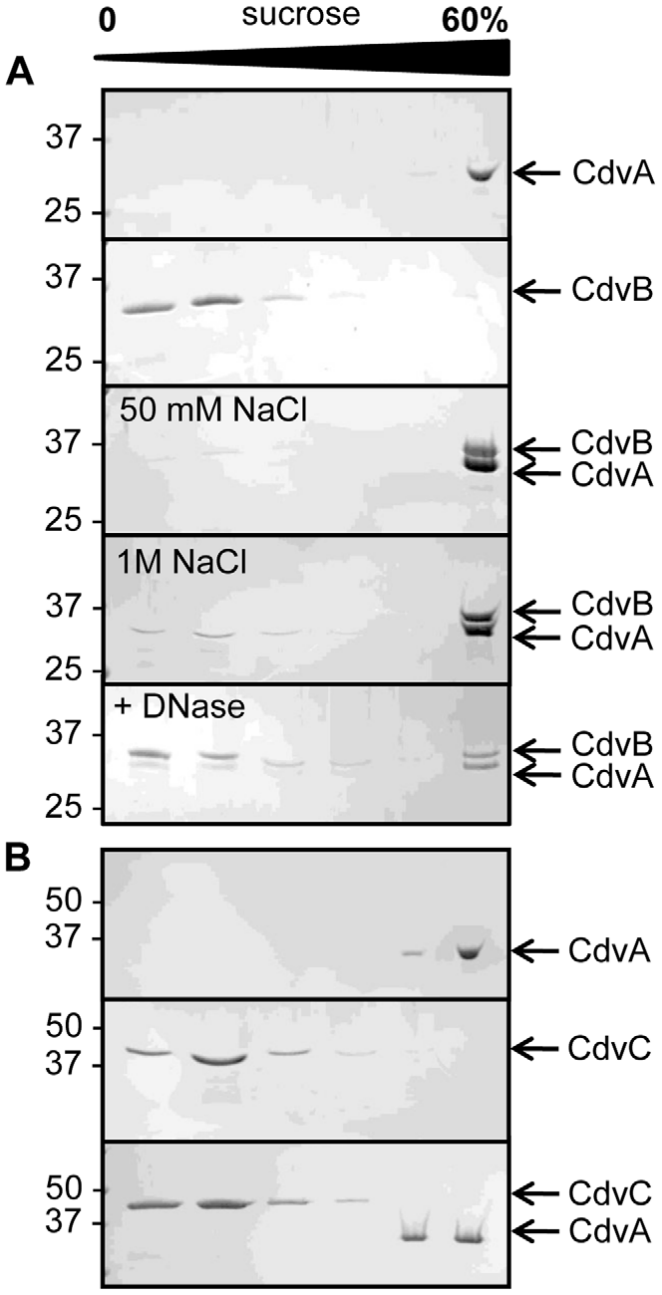
Interactions of *M. sedula* CdvA with CdvB or CdvC. (A) CdvA and CdvB (10 µM each) were incubated overnight at 4°C and the protein distribution of the mixed sample was analyzed by sucrose density gradient centrifugation (three lower panels) in comparison to single proteins (two upper panels). The sucrose gradient contained either 50 mM or 1 M NaCl, as specified on the graph. In the lower panel, CdvA was pretreated with 20 µg/ml DNase before incubation with CdvB in presence of 50 mM NaCl. Proteins were separated on a 12% SDS-PAGE and stained with Commassie blue. Values on the left correspond to the molecular weights of the marker proteins. (B) Similar experiment as in A with CdvA or CdvC alone (upper and middle panels) or the preincubated protein mixture (lower panel) resolved on a sucrose gradient containing 50 mM NaCl. The images are representative of at least 3 different experiments carried out with different batches of purified proteins.

To further explore interactions among the three Cdv proteins, we analyzed their co-migration during sucrose density gradient ultracentrifugation. While large CdvA-DNA oligomers were detected in the lower fractions of sucrose density gradient, full-length His-tagged CdvB was found in the upper fractions, in agreement with the fact that the latter was purified as a soluble and monodisperse protein (Figure 3A). In contrast, when incubated together, both proteins migrated in the 60% sucrose fraction. A similar profile was obtained when 1 M NaCl was added in the sucrose layers. Moreover, the interaction was still observed after treatment of CdvA with DNase. This indicates that CdvB interacts specifically with CdvA *in vitro*, a result consistent with the recent findings on interaction between the C-terminal region of CdvA and the wH domain of CdvB [25]. Moreover, we observed that the presence of CdvB did not change the appearance of the CdvA polymers (Figure S2). The same approach was applied to determine if CdvA also interacts with CdvC. As illustrated in Figure 3B, CdvC does not shift to higher sucrose densities when incubated in the presence of CdvA, indicating that no interaction occurs.

### CdvB shares structural similarity with ESCRT-III proteins and interacts with CdvC

**Figure 4:**
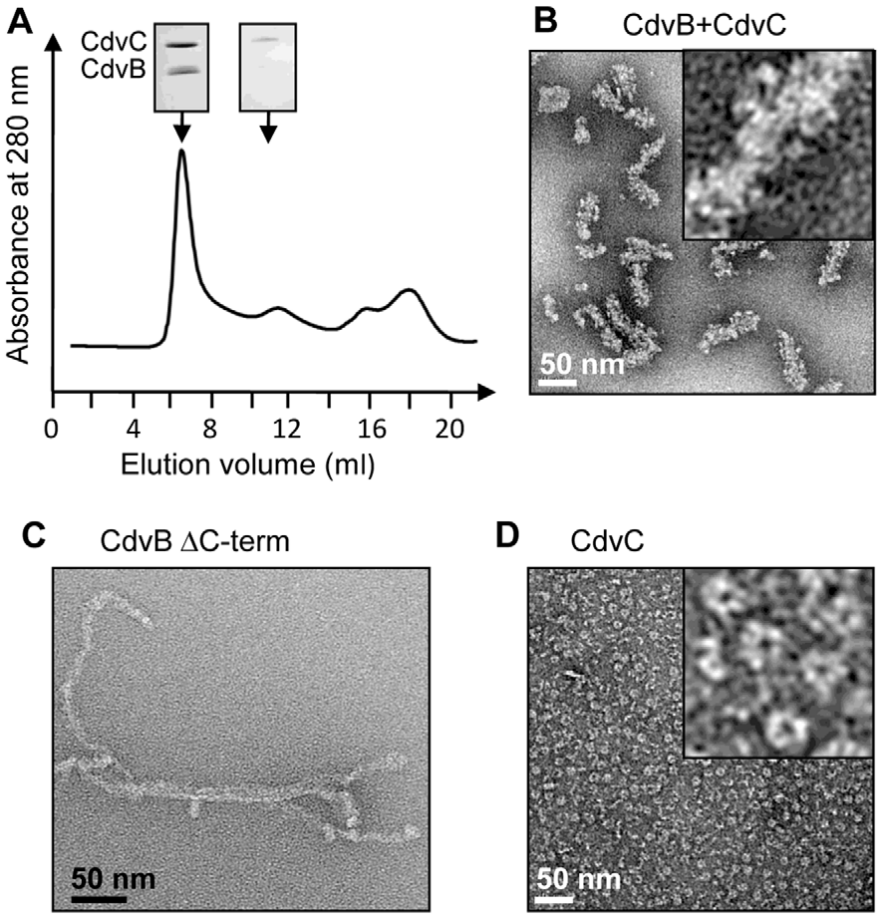
Structural analysis of *M. sedula* CdvB and CdvC homo and hetero-polymers. (A) SEC elution profile (Superose 6) of CdvB and CdvC mixture (20 µM each). Aliquots of the column fractions were separated on 12% SDS-PAGE showing that the 7 ml void volume peak contained both proteins while the excess of CdvC was found in the 12 ml peak. (B) Negative staining EM image of the CdvB-CdvC polymers. (C) Negative staining EM image of the CdvB deletion mutant (CdvΔC). (D) Negative staining EM image of CdvC before incubation with CdvB. The insets in B and C correspond to higher magnification (×5).

We then examined the interaction between CdvB and CdvC. The data obtained confirm that the *M. sedula* Vps4-like protein binds the ESCRT-III like subunit encoded by the adjacent gene, as previously shown for *S. solfataricus* [12] and *S. acidocaldarius* [10] (Figure S4). Importantly, we observed that the co-incubation of both proteins resulted in the formation of high molecular weight polymers which eluted in the void volume from a SEC column (Figure 4A). SDS-PAGE analysis confirmed that these large polymers contain CdvC as well as CdvB which both elute later when analyzed separately (Figure 1). Moreover, the intensity of the bands suggested the presence of equal amounts of CdvB and CdvC, indicating a potential 1∶1 stoichiometry (Figure 4A, inset). Negative staining EM revealed filamentous structures with variable diameters of 13±1 nm and 30+/−2 nm when decorated with CdvC (Figure 4B); however, we cannot rigorously exclude the possibility that these structures are aggregates. As expected, C-terminally truncated CdvB (CdvBΔC; residues 1–167) in which the MIM2 sequence required for interaction with CdvC is lacking [10], [27] no longer interacts with the latter (Figure S5). Interestingly, in contrast to what was observed with the full-length protein, CdvBΔC spontaneously formed curved filament-like structures (Figure 4C).

### The recombinant CdvC is an enzymatically active homopolymer

EM analysis of the CdvC homo-oligomers shows distinct polymers of various shapes, including ring-like particles exhibiting six- to seven-fold symmetry and a central pore (Figure 4D), as reported for mammalian homologous proteins [28], [29], [30]. Together with the molecular weight of the oligomers determined by MALLS (530±50 kDa complexes), these data indicate that CdvC may form dodecamers or tetradecamers which are most likely composed of two stacked rings. In agreement with the presence of typical Walker A (138-GPPGCGKT-145) and B (198-IIFIDE-303) ATPase motifs, we demonstrate that the CdvC oligomer has ATPase activity. In the presence of 2 mM ATP, the maximal CdvC ATPase activity was detected at 60°C with a rate of ATP hydrolysis about 0.5 µmol/min/mg protein (k_cat_ = 0.35 s^−1^) (Figure S6). This might not correspond to the optimal conditions for archaeal CdvC but CdvC precipitated at higher temperatures, thus impairing further measurements. Similarly, we were not able to test whether CdvC is able to disassemble the CdvB filaments shown in Figure 4B upon addition of ATP and Mg^2+^. This assay had to be performed at 60°C, because CdvC did not show any substantial ATPase activity at room temperature; however, the CdvB-CdvC filaments precipitated at elevated temperatures rendering it impossible to determine CdvB disassembly.

### The interactions of CdvA and CdvC with CdvB are not mutually exclusive

**Figure 5:**
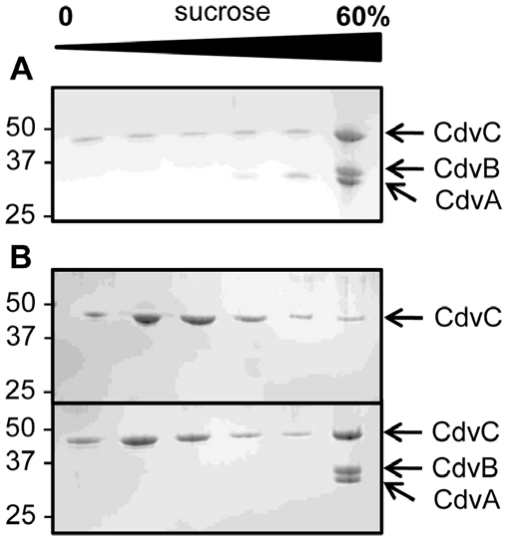
Concomitant interaction of *M. sedula* CdvB with both CdvA and CdvC. (A) CdvA, CdvB and CdvC (10 µM final concentration each) were incubated overnight at 4°C and the protein distribution of the mixed sample was analyzed by sucrose density gradient centrifugation. (B) The CdvA/CdvB complex was purified from the 60% sucrose fraction as shown in Figure 3 (panel A). This complex was then incubated with CdvC (10 µM final concentration) and analyzed by sucrose gradient centrifugation thus confirming results obtained when the 3 proteins were incubated together.

As shown by S. Bell's group, CdvB possess binding sites specific for CdvA and CdvC [10], [25]. Because these domains are located at the C-terminal end of Cdv B (Figure 1A), we investigated whether CdvA/CdvB and CdvC/CdvB interactions are mutually exclusive. The interaction between the three Cdv proteins was examined by sucrose density gradient ultracentrifugation of a mixture containing equal amount of CdvA, CdvB and CdvC. Alternatively, the CdvA/CdvB complex was purified and then incubated with CdvC before sucrose gradient centrifugation. The binding pattern of resulting interactions was identical in both experiments (Figure 5), demonstrating that binding of CdvA or CdvC to CdvB is clearly not mutually exclusive.

## Discussion

Here, we report the purification and biochemical characterization of the archaeal cell division proteins CdvA, CdvB and CdvC and the analysis of their interactions *in vitro*. Our first attempts to purify them from several crenarchaeal species were unsuccessful because the proteins were either poorly expressed or insoluble. The choice of the species *M. sedula* was critical in the success to purify soluble proteins from the *cdv* three-gene cluster.

Interestingly, we show that CdvA organizes into a double-helical structure containing DNA. Removal of DNA by DNase treatment destabilizes the polymers and produces a mixture of monomers and smaller polymers which have thinner appearance by negative staining EM, suggesting that the double-helical filaments correspond to CdvA polymers stabilized by DNA. Sequence analysis of CdvA did not reveal any conventional DNA-binding motifs. However, in accordance with previous reports on the homologous protein from *S. acidocaldarius* [9], the central region of the CdvA (residues 69–196) was found to possess the signatures of a coiled coil motif. Interestingly, the coiled coil domain of mammalian structural maintenance of chromosomes (SMC) proteins was previously implicated in DNA binding [31], although molecular details of this interaction are not known. We therefore hypothesize that the coiled coil region of CdvA might be responsible for the DNA binding and eventual formation of nucleoprotein filaments, which could be mediated by any of the 29 positively charged residues present in this region. The *in silico* analysis of CdvA also revealed that the beta-strand-rich N-terminal domain of this protein (residues 1–67) shares primary and secondary structure similarity with the photoreaction center (PRC)-barrel [32] or with MTH1859-like proteins [33] (PFAM ID: PF05239). Based on these structural similarities, a 3D model for this region of CdvA was proposed (Figure S7). The model was found to be most similar to the X-ray structure of the PRC-barrel protein MTH1859 from *Methanobacterium thermoautotrophicum* (PDB ID: 1PM3; [33] ) which belongs to a conserved family of proteins thought to function as adapters for the assembly of hetero-oligomeric complexes [33]. Interestingly, we noticed that in *Candidatus* Korarchaeum cryptofilum, a MTH1859-like protein is encoded in a seven gene cluster together with five paralogous *ftsZ* genes [34], suggesting that MTH1859-like adapter proteins might also play a role in FtsZ-dependent cell division.

The helical CdvA filaments morphologically resemble filaments formed *in vitro* by some bacterial DNA partitioning proteins including the tubulin/FtsZ-like protein TubZ [35], the actin-like ParM [36], or ParA2 [37]. Whereas some of these proteins self-assemble in an ATP-dependent manner [38], a DNA-dependent oligomerization was reported for ParA2 [37]. Thus the DNA-dependent polymerization of CdvA resembles most closely that of ParA2. Interestingly, the cytomotive cytoskeleton proteins which are involved in DNA or in organelle movement [39] are present in all bacteria and Archaea except Crenarchaeota [40]. However CdvA shows no significant sequence homology with known DNA segregation proteins. We also did not detect any sequence relationship to components of the eukaryotic cytoskeleton and nuclear envelope such as lamins, golgins and cingulin-like proteins, as previously reported [9]. Interestingly, a homologue of eukaryotic actin has been identified in Crenarchaeota lacking the three Cdv proteins (Thermoproteales) [24] and we noticed that actin is also present in a newly described archaeon [41] which harbours CdvB and CdvC, but not CdvA. Because the double helical structure of CdvA filaments resembles actin filaments, CdvA could be a functional analogue of archaeal actin. Thus both the morphology of CdvA filaments and the phylogenetic analyses support the hypothesis that it constitutes an ancient cytoskeleton protein involved in cell division. Moreover, the formation of such helical filaments fit well with observations of CdvA structures in the cell prior to nucleoid segregation [25].

Another main finding is that, in contrast with the full-length CdvB (the archaeal homologue of ESCRT-III proteins) which was purified as a monodisperse protein, its C-terminus truncated version polymerizes *in vitro* and forms filaments, which is reminiscent of eukaryotic ESCRT-III proteins [42], [43], [44], [45]. A common feature of eukaryotic ESCRT-III proteins is that they shuttle between an autoinhibited inactive conformation in the cytosol and an active membrane-bound conformation; activation entails displacement of a C-terminal region from the N-terminal core, which can be mimicked by C-terminal deletions [46], [47], [48], [49], [50], [51]. Because such polymers have been implicated in membrane fission [14], [17], [46], [52]. CdvB polymers might exert the same function during cell division, which is closely controlled by CdvC that is present in ring-like structures throughout the whole division stage [53].

Finally, SEC and MALLS analysis indicate that CdvC, the third member of the crenarcheal cell division machinery homologous to eukaryotic Vps4, might form mostly dodecamers, although preliminary single particle EM analysis indicates the presence of ring-like structures with six and seven-fold symmetry. One main function of eukaryotic Vps4 is ESCRT-III disassembly via its ATPase activity [17], [22], as confirmed *in vitro* [43], [54]. This requires the nucleotide dependent oligomerization of Vps4 into dodecamers [28], [29], [30], [55] or potential tetradecamers [30]. While eukaryotic Vps4 only forms double ring-like structures in the presence of nucleotides, the archaeal CdvC formed them efficiently already in the absence of nucleotides in a concentration dependent manner. Notably, the SEC profile did not change in the presence of ATP or AMP-PNP. Moreover, we observed that dodecameric/tetradecameric CdvC efficiently hydrolyzes ATP while the *S. solfataricus* dimer was found to be inactive under the conditions tested [11]. Although the CdvC polymers were active, we could not measure depolymerization of CdvB filaments because they precipitated under the experimental conditions required for CdvC ATPase activity.

CdvA functions together with CdvB and CdvC during archaeal division [9], [10]. By sucrose density gradient ultracentrifugation, we confirmed that CdvA physically interacts with CdvB, a point recently evidenced by using other approaches [25]. In contrast, we showed that CdvA does not interact with CdvC that recruits CdvB via its N-terminus MIT domain [10], [12]. With two potential interaction partners, CdvB could exert a pivotal role in the cell division process. The binding sites identified to be necessary for its interaction with CdvA and CdvC are both located within the C-terminal region [10], [25], suggesting that binding of proteins to these regions may be mutually exclusive. Here we showed that both CdvA and CdvC can interact at the same time with CdvB.

In summary, our structural and biochemical analysis of CdvA, CdvB and CdvC proteins from *M. sedula* provides further insight into the crenarchaeal ESCRT-III/Vps4 system and reveals its association with a unique and novel type of archaeal cytomotive cytoskeleton protein. We hypothesize that CdvA first binds chromosomal DNA, possibly in association with other segregation proteins, such as ParA and/or SMC proteins. In particular, CdvA might play an important role in the formation of a synaptic-like structure bridging the two chromosomes in Sulfolobales during the G2 phase of the cell cycle that precedes mitosis [56]. The CdvA/DNA complex could then recruit the CdvB subunits between segregating nucleoids where their polymerization could take place. Importantly, the mitotic localization of CHMP3 and CHMP4B to kinetochores and at spindle fibers was recently demonstrated [57] as well as the functional role of both ESCRT-III and Vps4 proteins in the mammalian centrosomes/spindle maintenance [19]. From these observations, it has been suggested that, in addition to its role in membrane fission, the ESCRT-III machinery might organize the coordination of the three main steps during cell division, i.e. centrosome duplication, chromosome segregation and cytokinesis [19]. Thus the close link between CdvA and CdvB/C suggests a similar scenario for crenarchaeal cell division.

## Materials and Methods

### Cloning of cvd genes into expression vector

The genes from the cdv operon were amplified by PCR from genomic DNA of *M. sedula* using the High-Fidelity PCR master mix (Phusion®, Finnzymes) and gene-specific primers encoding a TEV site (Table S1). The resulting PCR products were cloned into the pDEST-17 vector using the Gateway cloning system from Invitrogen and following the manufacturer's instructions. The integrity of all constructs was confirmed by DNA sequencing.

### Expression and purification of Cdv proteins

The expression of recombinant Cdv proteins was carried out *in E.coli* C41(DE3). Transformed cells were grown in LB^amp^ medium to an absorbance of approximately 0.5 and expression was induced for 1 hour at 37°C by addition of IPTG to a final concentration of 0.1 mM. The cells were then pelleted by centrifugation (5,000×*g*, 20 min) and resuspended in lysis buffer before sonication. In the optimized CdvA purification protocol, following sonication (Tris 50 mM pH 8.8, NaCl 350 mM, Triton 0.2% and Complete Protease Inhibitor® (Roche Diagnostics)), the bacterial lysate was heated for 30 min at 75°C and centrifuged (20,000 rpm, 30 min, 4°C). The supernatant supplemented with 10 mM imidazole, 50 mM glutamate and 50 mM L-arginine was then applied onto a Ni^2+^-NTA column (Qiagen) for affinity purification. After extensive washings (Tris 50 mM pH 8.8, NaCl 350 mM, glutamate 50 mM, L-argininine 50 mM, imidazole 20 mM, then 50 mM) the protein was eluted in presence of 300 mM imidazole.

Lysis of CdvB- or CdvC-expressing bacteria was performed in Tris 50 mM pH 8.8, NaCl 50 mM, CHAPS 1%, 10 mM imidazole and Complete Protease Inhibitor®, and the resulting supernatant was directly loaded onto a Ni^2+^-NTA column without heating step. The eluted protein was further purified by gel filtration (Superdex 200 for CdvB or Superose 6 for CdvC) using Tris 50 mM pH 8.8, NaCl 50 mM as buffer. The same protocol was applied for CdvB and CdvBΔC-term except than a higher NaCl concentration (350 mM) was required. Gel filtration was performed on AKTA Purifier (GE Healthcare) at 0.5 ml/min flow rate. In all cases, the purity and integrity of the fusion proteins were analyzed by 12% SDS-PAGE and Commassie blue staining.

### Ammonium sulfate precipitation

The CdvC eluate from the Ni^2+^-NTA column (total volume 25 ml) was divided in two equal fractions. The first one was directly concentrated in a small volume (700 µl at 289 µM), then purified on Superose 6 using Tris 50 mM pH 8.8, NaCl 50 mM as buffer. A saturated ammonium sulfate solution was added to the second fraction and the mixture was incubated on ice for 2 h and centrifuged at 16,000 for 30 min to recover the precipitated protein. The pellet was resuspended in 700 µl of Tris 50 mM pH 8.8, NaCl 50 mM, dialyzed to remove residual ammonium sulfate, and then passed to the Superose 6 column.

### Size exclusion chromatography combined with detection by multi-angle laser light scattering analysis

The heterogeneity of the sample was analyzed by SEC combined with detection by MALLS and refractometry [58]. SEC was performed with a Superose 6 column equilibrated with 50 mM Tris/HCl at pH 8.8 containing 50 mM NaCl using an HPLC system (Waters). Separations were performed at 20°C with a flow rate of 0.5 ml.min^−1^. Fifty to 400 µL of a CdvC solution at a concentration of 180 µM (8.2 mg.mL^−1^) were injected. On-line MALLS detection was performed with a DAWN-EOS detector (Wyatt Technology Corp., Santa Barbara, CA) using a laser emitting at 690 nm. Data were analyzed and absolute molecular masses (M_w_) were calculated using the ASTRA software (Wyatt Technology Corp., Santa Barbara, CA) as described previously [59].

### Electron microscopy

For negative staining, the samples were adsorbed to the clean side of a carbon film on mica, stained with 1% sodium silicotungstate pH 7.5 and transferred to a 400-mesh copper grid. The images were taken under low dose conditions at a nominal magnification of 45,000 times with defocus values between 0.9 and 1.5 µm on a Philips CM12 LaB6 electron microscope at 120 kV accelerating voltage and Orius SC1000 CCD GATAN CCD camera.

### Sucrose gradient density ultracentrifugation

A discontinuous sucrose density gradient was prepared manually by layering upon one another successive decreasing sucrose density solutions ranging from 60 to 10% (w/v) in Tris 50 mM pH 8.8 NaCl 50 mM. In some experiments, as indicated in the result section, the concentration of NaCl was of 1 M. Proteins or protein mixtures were then applied on the top and the tubes were submitted to ultracentrifugation in a swinging bucket SW 55Ti rotor (Beckman) at 40,000 rpm (RCFav 132 000×*g*) for 5 hours 4°C. Immediately after the run, the tubes were carefully removed from the rotor and proteins present in each layer were analyzed by SDS-PAGE.

### Detection of ATPase activity

ATPase activities were performed using a coupled enzymatic assay containing pyruvate kinase and lactate dehydrogenase as previously described [60], and with temperatures ranging from 37°C to 65°C. Hence, one mole of ATP hydrolysed is directly converted to one mole of NADH oxidized to NAD^+^, and the ATPase activity was monitored at 60°C by the disappearance of NADH followed at OD340 nm using a Safas UVmc^2^ spectrophotometer. A typical reaction mixture (1 ml) contained 50 mM Tris/HCl, pH 8.0, 30 mM KCl, 4 mM PEP, 60 µg/ml PK, 32 µg/ml LDH, 0.4 mM NADH, 5 mM MgCl_2_, 2 mM ATP and various amounts of proteins.

### Sequence analysis

Homology searches of CdvA, CdvB and CdvC were performed by Blastp and PSI-Blast at the nr and environmental databases at the NCBI (http://www.ncbi.nlm.nih.gov/). Domain searches were performed at http://pfam.sanger.ac.uk/search. Multiple alignments were obtained by using Muscle [61]. Phylogenetic analysis was performed on a selection of unambiguously aligned amino acid positions by using Treefinder [62] with the LG+Γ model [63] with four rate categories, which was selected as the best-fit model for all our individual and concatenated data sets by the model selection tool implemented in TREEFINDER. Node support was assessed by 100 bootstrap replicates with the same model.

### Structure modelling

Structure modelling was performed with I-TASSER, which uses a combination of *ab initio* and homology-based approaches for structural modelling [64] The X-ray structure of the putative adapter protein MTH1859 (PDB ID: 1PM3) from *Methanobacterium thermoautotrophicum* [33] was identified by I-TASSER as the best template and was subsequently used for structural modelling.The stereochemical quality was assessed using ProSA-web [65]. ProSA-web quality scores for the CdvA N-terminal domain was calculated to be −5.52 (supplemental Fig. S7). The closest structural homologue of the CdvA N-terminal domain was identified using Vector Alignment Search Tool [66]. Coiled coil domain was identified using COILS [67] at http://www.ch.embnet.org/software/COILS_form.html.

## Supporting Information

Figure S1 **Elution profile of CdvC from a Superose 6 SEC column.** The dashed line (—) represents the gel filtration profile of the CdvC eluate collected from the Ni^2+^-NTA column and concentrated (389 µM). The dotted (- - -) line represents the profile of another aliquot of the same CdvC eluate precipitated with ammonium sulfate and dialyzed before SEC analysis (350 µM).
(TIF)

Figure S2 **EM images of CdvA filaments formed in presence of different compounds.** Scale bar : 50 nm.
(TIF)

Figure S3 **Maximum likelihood tree of CdvA homologues.** The tree is rooted in between Thaumarchaeota and Crenarchaeota. Thaumarchaeota are indicated in blue font, Sulfolobales in orange font, and Desulfurococcales in pink font. The arrow indicates the protein of *M. sedula* studied here. Scale bar represents the average number of substitutions per site. Numbers at nodes represent bootstrap proportions.
(TIF)

Figure S4 **Interaction of** ***M. sedula*** **CdvB with CdvC.** CdvB and CdvC (10 µM each) were incubated overnight at 4°C and the protein distribution of the mixed sample was analyzed by sucrose density gradient centrifugation (lower panels) in comparison with pure proteins (two upper panels) in presence of 50 mM NaCl. Proteins were visualized by separation on a (12%) SDS-PAGE stained with Commassie blue. Values on the left correspond to the molecular weights of the marker proteins.
(TIF)

Figure S5 **Interaction of** ***M. sedula*** **CdvBΔC with CdvC.** Same legend as in Figure S4 with a truncated form of CdvB.
(TIF)

Figure S6 **ATPase activity of CdvC.** The ATPase activity of *M. sedula* CdvC was monitored at 60°C by following the disappearance of NADH at 340 nm using a coupled enzymatic assay (see Materials and Methods). The assay contained 8 µg (green line), 16 µg (blue line) or 32 µg (red line) of the purified protein. A control performed in the absence of protein but in the presence of ATP is also shown (grey line) corresponding to the spontaneous hydrolysis of ATP at this temperature. In the absence of ATP but in the presence of the same amounts of CdvC, no NADH disappearance was observed. This picture is representative of three independent experiments.
(TIF)

Figure S7 **Three dimensional model of the N-terminal domain of CdvA (residues 1–67).** (A) Comparison of the three dimensional model of the CdvA N-terminal domain with X-ray structures of the PRC-barrel proteins, which are indicated by their PDB identifiers (3HTR, PRC-barrel domain protein from *Rhodopseudomonas palustris*; 1PM3, putative adapter protein MTH1859 from *Methanobacterium thermoautotrophicum*; 1AIG, chain H, photosynthetic reaction center from *Rhodobacter sphaeroides*). The structures are colored using a gradient from red (N-terminus) to blue (C-terminus) color scheme according to the position within the structural alignment, so that equivalent positions are colored similarly. Residues 1–141 of 1AIG as well as residues 82–120 of 3HTR have been omitted for more convenient representations. (B) Structure-based sequence alignment of proteins is shown in Figure S7A. The alignment is colored according to sequence conservation (BLOSUM62 matrix). (C) Quality assessment of the three-dimensional model. Quality of the generated model along with that of structural homologues shown in Figure S7A was evaluated using PsoSA-web at https://prosa.services.came.sbg.ac.at/prosa.php. The quality (Z) score of the model (CdvA_N; Z = −5.52) is displayed in the context of the Z-scores of all experimentally determined protein structures available in the Protein Data Bank. Every dot represents a distinct structure solved by X-ray crystallography (light blue) or NMR (dark blue).
(TIF)

Table S1 **List of primers used for cloning.**
(DOC)
