# Supplementary figures and images for: Crenarchaeal CdvA Forms Double-Helical Filaments Containing DNA and Interacts with ESCRT-III-Like CdvB
Source: PLoS One. 2011 Jul 8;6(7):e21921. doi: 10.1371/journal.pone.0021921 (PMC3132758; doi:10.1371/journal.pone.0021921)

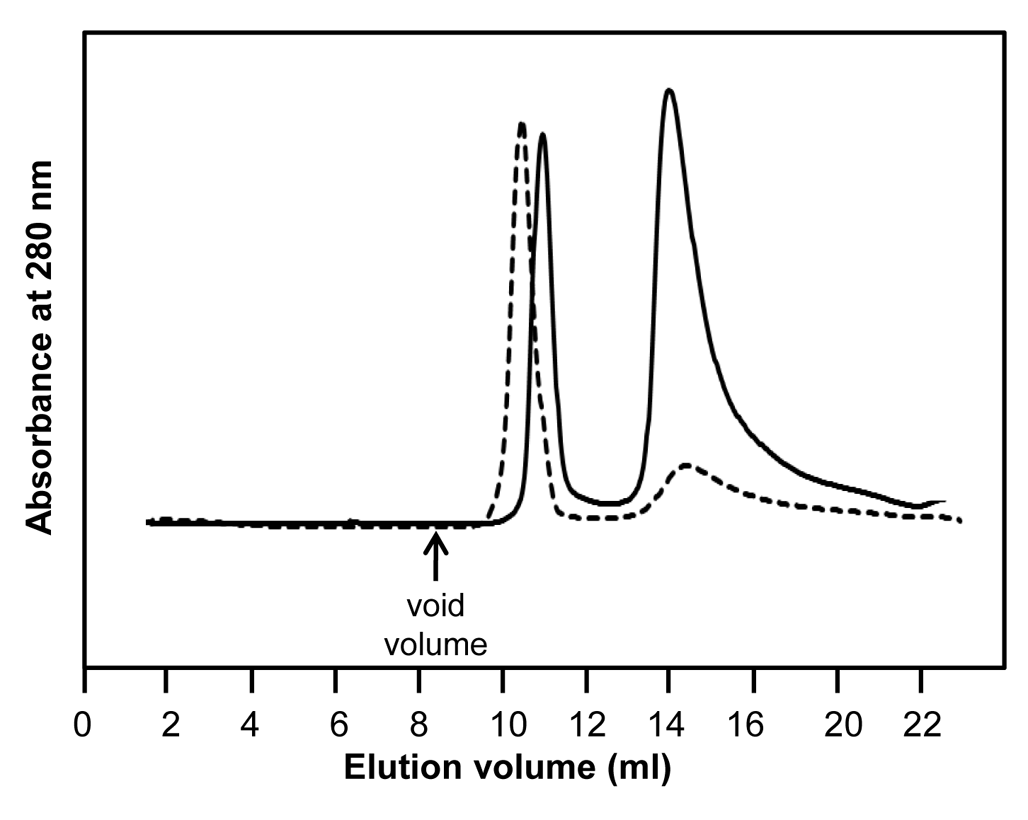

Supplement: Figure S1 — Elution profile of CdvC from a Superose 6 SEC column. The dashed line (—) represents the gel filtration profile of the CdvC eluate collected from the Ni2+-NTA column and concentrated (389 µM). The dotted (- - -) line represents the profile of another aliquot of the same CdvC eluate precipitated with ammonium sulfate and dialyzed before SEC analysis (350 µM). (TIF) [file pone.0021921.s001.tif]

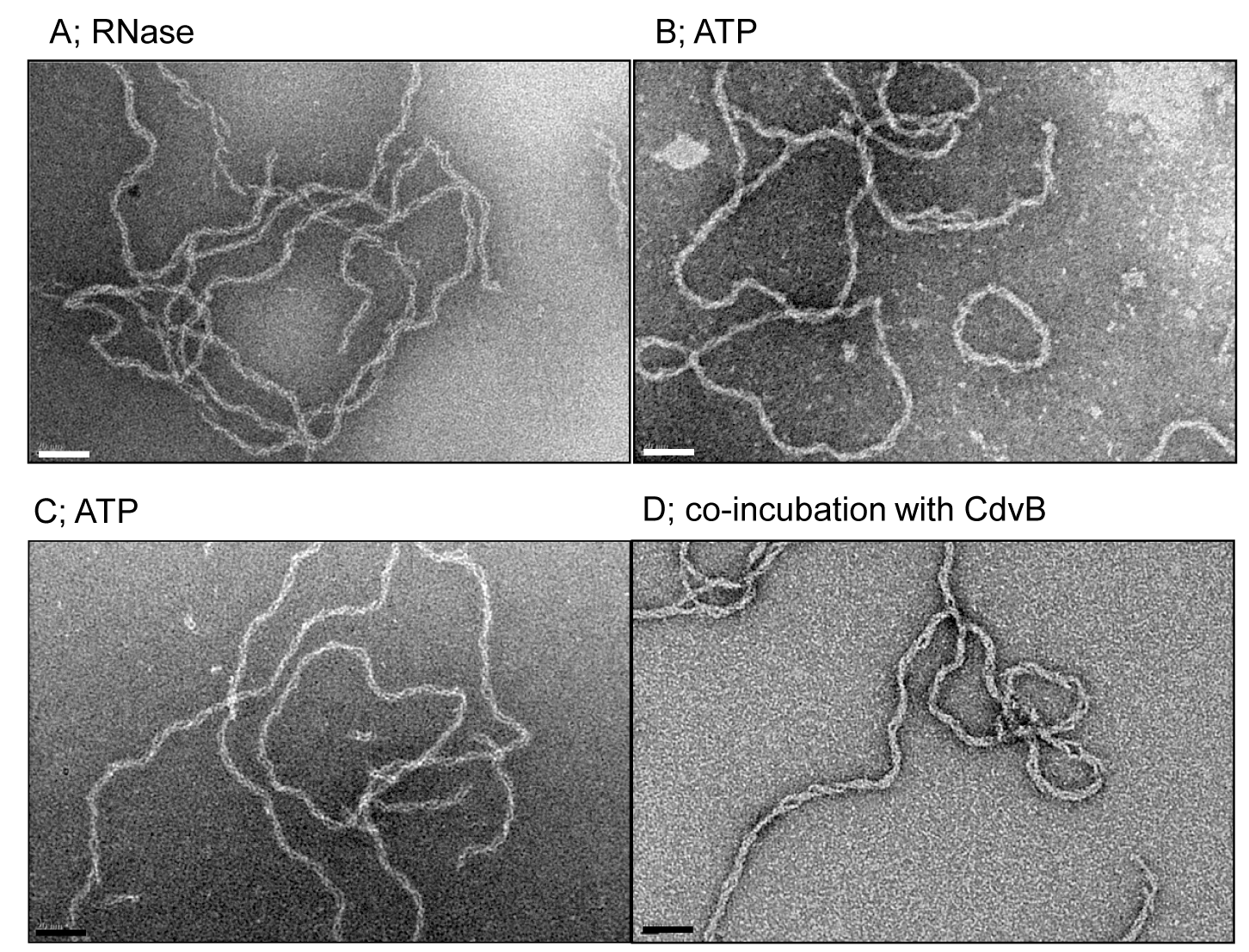

Supplement: Figure S2 — EM images of CdvA filaments formed in presence of different compounds. Scale bar : 50 nm. (TIF) [file pone.0021921.s002.tif]

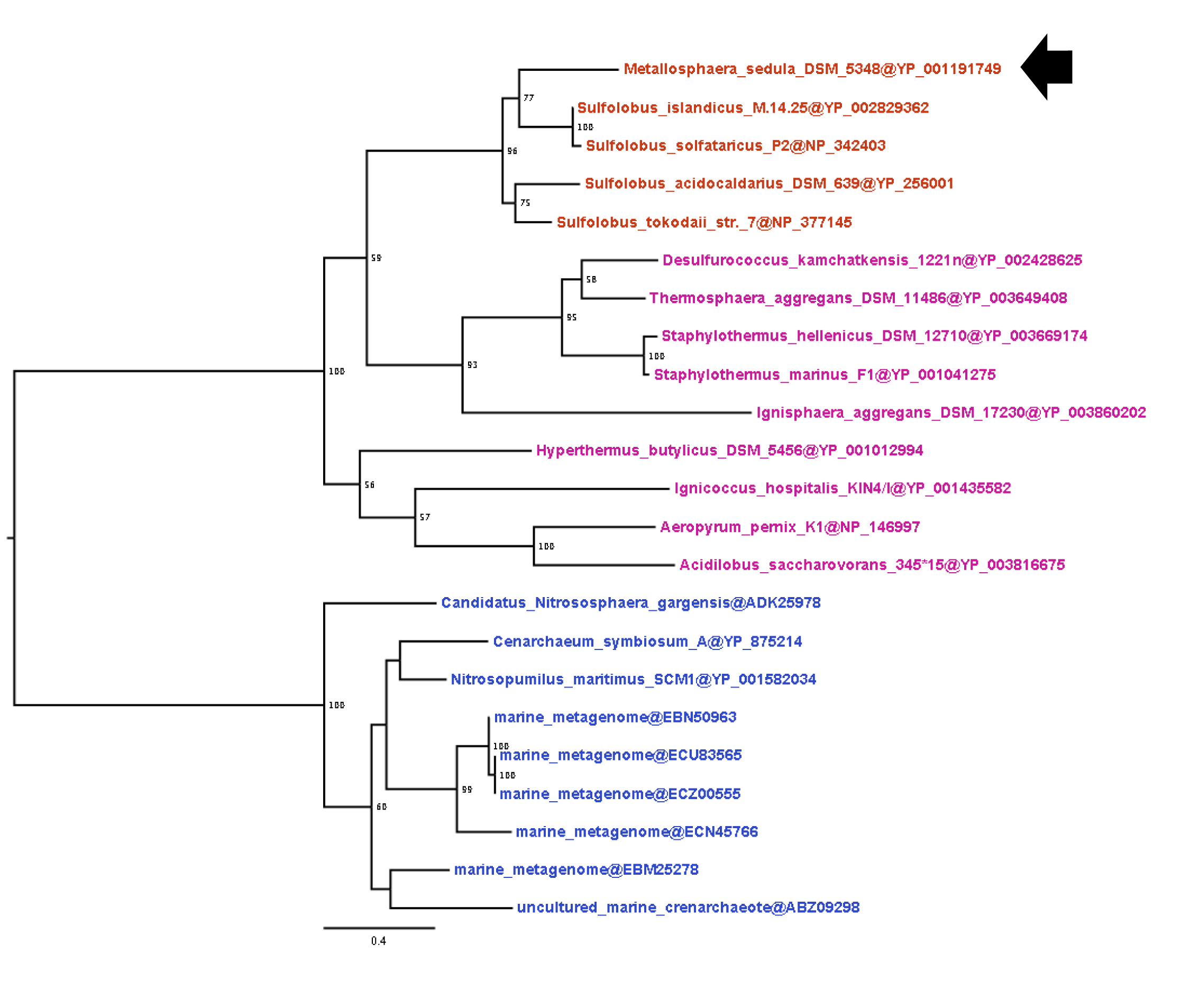

Supplement: Figure S3 — Maximum likelihood tree of CdvA homologues. The tree is rooted in between Thaumarchaeota and Crenarchaeota. Thaumarchaeota are indicated in blue font, Sulfolobales in orange font, and Desulfurococcales in pink font. The arrow indicates the protein of M. sedula studied here. Scale bar represents the average number of substitutions per site. Numbers at nodes represent bootstrap proportions. (TIF) [file pone.0021921.s003.tif]

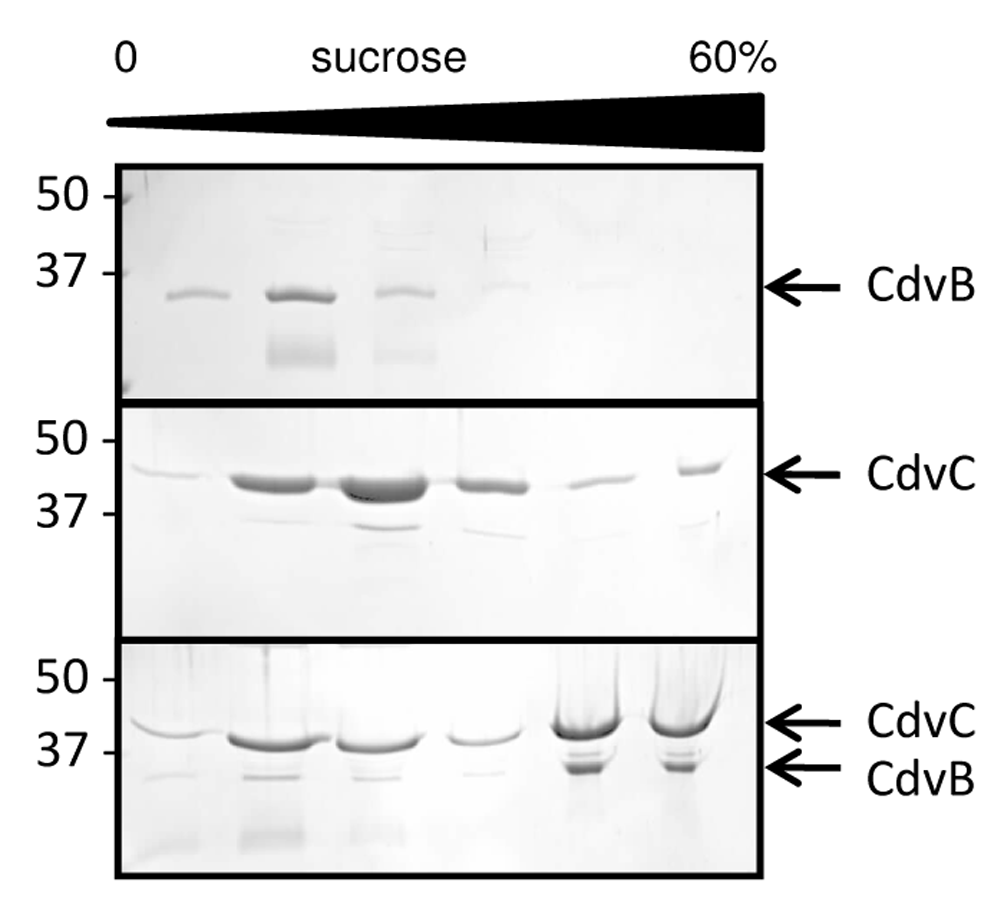

Supplement: Figure S4 — Interaction of M. sedula CdvB with CdvC. CdvB and CdvC (10 µM each) were incubated overnight at 4°C and the protein distribution of the mixed sample was analyzed by sucrose density gradient centrifugation (lower panels) in comparison with pure proteins (two upper panels) in presence of 50 mM NaCl. Proteins were visualized by separation on a (12%) SDS-PAGE stained with Commassie blue. Values on the left correspond to the molecular weights of the marker proteins. (TIF) [file pone.0021921.s004.tif]

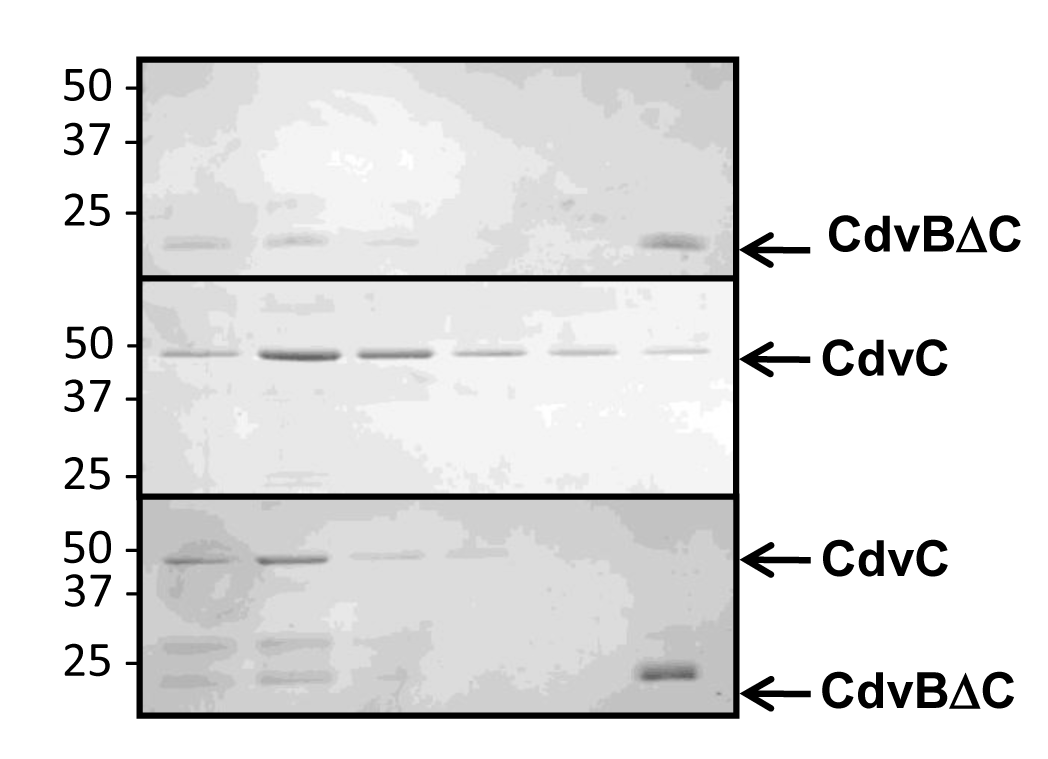

Supplement: Figure S5 — Interaction of M. sedula CdvBΔC with CdvC. Same legend as in Figure S4 with a truncated form of CdvB. (TIF) [file pone.0021921.s005.tif]

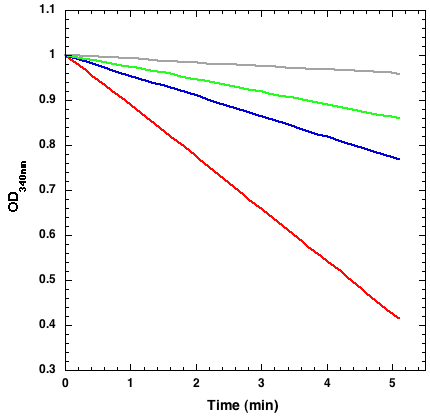

Supplement: Figure S6 — ATPase activity of CdvC. The ATPase activity of M. sedula CdvC was monitored at 60°C by following the disappearance of NADH at 340 nm using a coupled enzymatic assay (see Materials and Methods). The assay contained 8 µg (green line), 16 µg (blue line) or 32 µg (red line) of the purified protein. A control performed in the absence of protein but in the presence of ATP is also shown (grey line) corresponding to the spontaneous hydrolysis of ATP at this temperature. In the absence of ATP but in the presence of the same amounts of CdvC, no NADH disappearance was observed. This picture is representative of three independent experiments. (TIF) [file pone.0021921.s006.tif]

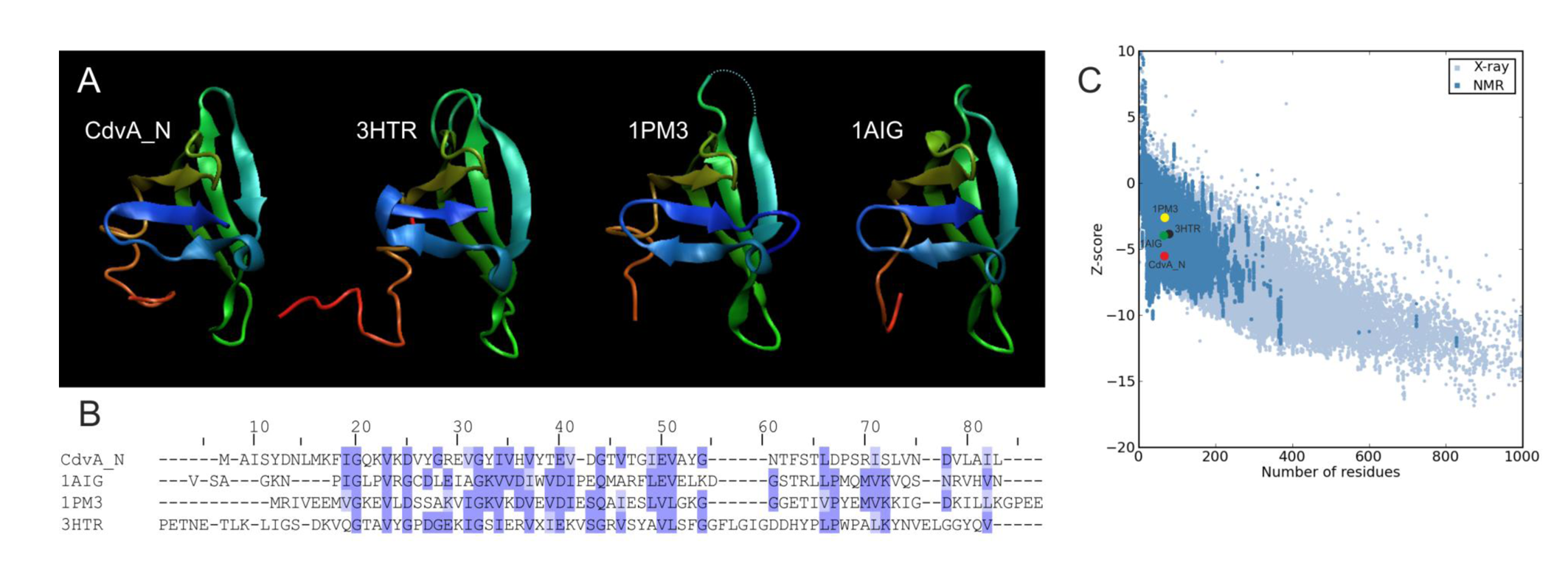

Supplement: Figure S7 — Three dimensional model of the N-terminal domain of CdvA (residues 1–67). (A) Comparison of the three dimensional model of the CdvA N-terminal domain with X-ray structures of the PRC-barrel proteins, which are indicated by their PDB identifiers (3HTR, PRC-barrel domain protein from Rhodopseudomonas palustris; 1PM3, putative adapter protein MTH1859 from Methanobacterium thermoautotrophicum; 1AIG, chain H, photosynthetic reaction center from Rhodobacter sphaeroides). The structures are colored using a gradient from red (N-terminus) to blue (C-terminus) color scheme according to the position within the structural alignment, so that equivalent positions are colored similarly. Residues 1–141 of 1AIG as well as residues 82–120 of 3HTR have been omitted for more convenient representations. (B) Structure-based sequence alignment of proteins is shown in Figure S7A. The alignment is colored according to sequence conservation (BLOSUM62 matrix). (C) Quality assessment of the three-dimensional model. Quality of the generated model along with that of structural homologues shown in Figure S7A was evaluated using PsoSA-web at https://prosa.services.came.sbg.ac.at/prosa.php. The quality (Z) score of the model (CdvA_N; Z = −5.52) is displayed in the context of the Z-scores of all experimentally determined protein structures available in the Protein Data Bank. Every dot represents a distinct structure solved by X-ray crystallography (light blue) or NMR (dark blue). (TIF) [file pone.0021921.s007.tif]
