# Supplementary material for: Crenarchaeal CdvA Forms Double-Helical Filaments Containing DNA and Interacts with ESCRT-III-Like CdvB
Source: PLoS One. 2011 Jul 8;6(7):e21921. doi: 10.1371/journal.pone.0021921 (PMC3132758; doi:10.1371/journal.pone.0021921)
Supplement: Table S1 — List of primers used for cloning. (DOC) [file pone.0021921.s008.doc]

**Table S1. List of primers used for cloning**

| **Protein** | **gene** |  | **Primer sequences** |  |  |
| --- | --- | --- | --- | --- | --- |
| CdvA | Msed_1670 | F | GGGGACAAGTTTGTACAAAAAAGCAGGCTTAGAAAACCTGTACTTCCAGGGTGCTATATCGTATGATAATTTAATG | |  |
|  |  | R | GGGGACCACTTTGTACAAGAAAGCTGGGTCTTATTACTGTGTGCTTAAGACTTTAACAGG | |  |
| CdvB | Msed_1671 | F | GGGGACAAGTTTGTACAAAAAAGCAGGCTTAGAAAACCTGTACTTCCAGGGTAAGTTAAGCTCACTTTTCAAC | |  |
|  |  | R | GGGGACCACTTTGTACAAGAAAGCTGGGTCTTATTACGCCTCCAGTGCAATCAG |  |  |
| CdvBΔC-term | Msed_1671 | F | GGGGACAAGTTTGTACAAAAAAGCAGGCTTAGAAAACCTGTACTTCCAGGGTAAGTTAAGCTCACTTTTCAAC | |  |
|  |  | R | GGGGACCACTTTGTACAAGAAAGCTGGGTCTTATTACTCATCGTCCACTGTGGGGAC | |  |
| CdvC | Msed_1672 | F | GGGGACAAGTTTGTACAAAAAAGCAGGCTTAGAAAACCTGTACTTCCAGGGTAGTGCCCAAGTAATGCTCGAGG | |  |
|  |  | R | GGGGACCACTTTGTACAAGAAAGCTGGGTCTTATTATAATGCCTTAAACTTTTCGTGCC | |  |
|  |  |  |  |  |  |
